# Dropout in a longitudinal, cohort study of urologic disease in community men

**DOI:** 10.1186/1471-2288-6-58

**Published:** 2006-12-14

**Authors:** Naomi M Gades, Debra J Jacobson, Michaela E McGree, Michael M Lieber, Rosebud O Roberts, Cynthia J Girman, Steven J Jacobsen

**Affiliations:** 1 Division of Epidemiology, Department of Health Sciences Research, Mayo Clinic College of Medicine, Rochester, Minnesota, USA; 2 Division of Biostatistics, Department of Health Sciences Research, Mayo Clinic College of Medicine, Rochester, Minnesota, USA; 3 Department of Urology, Mayo Clinic College of Medicine, Rochester, Minnesota, USA; 4 Department of Epidemiology, Merck Research Laboratories, Blue Bell, Pennsylvania, USA; 5 Research and Development, Southern California Permanente Medical Group, Pasadena, California, USA

## Abstract

**Background:**

Reasons for attrition in studies vary, but may be a major concern in long-term studies if those who drop out differ systematically from those who continue to participate. Factors associated with dropout were evaluated in a twelve-year community-based, prospective cohort study of urologic disease in men.

**Methods:**

During 1989–1991, 2,115 randomly selected Caucasian men, ages 40–79 years from Olmsted County, Minnesota were enrolled and followed with questionnaires biennially; 332 men were added in follow-up. A random subset (~25%) received a urologic examination. Baseline characteristics including age, benign prostatic hyperplasia (BPH) symptoms, comorbidities, and socioeconomic factors were compared between subjects who did and did not participate after the twelfth year of follow-up.

**Results:**

Of the 2,447 men, 195 died and were excluded; 682 did not participate in 2002. Compared with men in the 40–49 year age group, men ≥ 70 years of age at baseline had a greater relative odds of dropout, 2.65 (95% CI: 1.93, 3.63). In age-adjusted analyses, relative to men without stroke, men who had suffered a stroke had a higher odds of dropout, age-adjusted OR 3.07 (95% CI: 1.49, 6.33). Presence of at least one BPH symptom was not associated with dropout, (age-adjusted OR 1.12 (95% CI: 0.93, 1.36)).

**Conclusion:**

These results provide assurance that dropout was not related to primary study outcomes. However, factors associated with dropout should be taken into account in analyses where they may be potential confounders.

## Background

Attrition becomes a major concern in long-term studies if those who drop out differ systematically from those who continue to participate. Common statistical methods used for cohort studies assume there is no difference between subjects who drop out of the study and those who continue to participate. However, if this assumption is false, the validity of the study may be jeopardized and the difference between the observed values and "true" values may increase with study follow-up [1]. This is of particular concern if disease severity influences study participation. Losses in long-term studies may be due to refusals to continue in a study, inability to trace individuals for follow-up, and deaths.

Few studies of urologic conditions, such as benign prostatic hyperplasia (BPH), have examined dropout of study participants over the long term. One prospective cohort study of urinary symptoms followed participants for up to four years, and documented a dropout rate of 29%. Men with the highest levels of lower urinary tract symptom bother and interference at baseline, dropped out by last year of follow-up [2]. Two long-term, double-blind clinical trials which evaluated urologic outcomes, the Medical Therapy of Prostatic Symptoms (MTOPS) trial [3] and the Proscar Long-term Efficacy and Safety Study (PLESS) [4], evaluated dropout. In the MTOPS study the rate of loss was 2.5 per 100 patient-years in the placebo group and ranged from 2.2 to 3.3 in the treatment groups [3]. By the end of the PLESS study, 42% of patients had discontinued treatment. However, follow-up data were available on 92% of the randomized patients, but differences in men who dropped out compared with those who continued to participate were not examined [5]. Roehrborn [6] examined the importance of patient follow-up after discontinuation and concluded that in order to compare the true event rate across clinical trials, discontinuation rates and total patient follow-up for clinical outcomes must be assessed, a recommendation that is applicable to cohort studies as well.

The Olmsted County Study of Urinary Symptoms and Health Status among Men is a community-based, prospective cohort study that offers an ideal opportunity to identify characteristics associated with dropout, and to determine if these factors might bias results from this study.

## Methods

### Study design and population

The Olmsted County Study of Urinary Symptoms and Health Status among Men was initiated in December 1989 and details have been published elsewhere [7-9]. Briefly, this study is a community-based, prospective cohort study of Caucasian men who were 40–79 years of age on January 1, 1990, randomly selected from the Olmsted County, Minnesota population. The goal of the study was to determine the natural history of urinary symptoms and BPH. Potential subjects were identified through the Rochester Epidemiology Project [10]. Community medical records of all the randomly selected men were reviewed for history of prostate cancer, prostatectomy, and other medical conditions which may impede normal voiding function, such as neurologic disease, lower back surgery and urethral stricture. After exclusion for these pre-existing conditions and/or treatments, 3,874 men were asked to join the study and of these 2,115 agreed to participate (55% participation rate).

### Data collection

Individual participants were visited in their homes, signed informed consent documentation, and were asked to complete a questionnaire which documented demographic information, including the participant's age, marital status, education level, and annual salary. The questionnaire also assessed lower urinary tract symptom severity with questions similar to the American Urological Association Symptom Index (AUASI) [11-13]. Urinary bother and interference with daily activities were assessed using a Health-Related Quality of Life (HRQL) questionnaire specific for BPH [14]. Information on the presence of comorbidities and other urologic conditions, including erectile dysfunction (ED), diabetes mellitus, stroke, heart disease, and hypertension were reported on the baseline questionnaire. In addition, men voided into a portable uroflow meter (Dantec 1000) to assess lower urinary function.

A 25% random sub-sample of the study cohort, 475 out of 537 men (88%), agreed to participate in a detailed clinical urologic examination, including serum prostate specific antigen (PSA) level, digital rectal examination, and transrectal sonographic imaging of the prostate to determine prostatic volume. Measurements were made of the anteroposterior, transverse, and superoinferior diameters and prostate volume was estimated using the formula for a prolate ellipsoid, π/6 (*transverse × anteroposterior × superoinferior*) [15]. All study procedures were approved by the Mayo Foundation Institutional Review Board (IRB).

**Figure 1:**
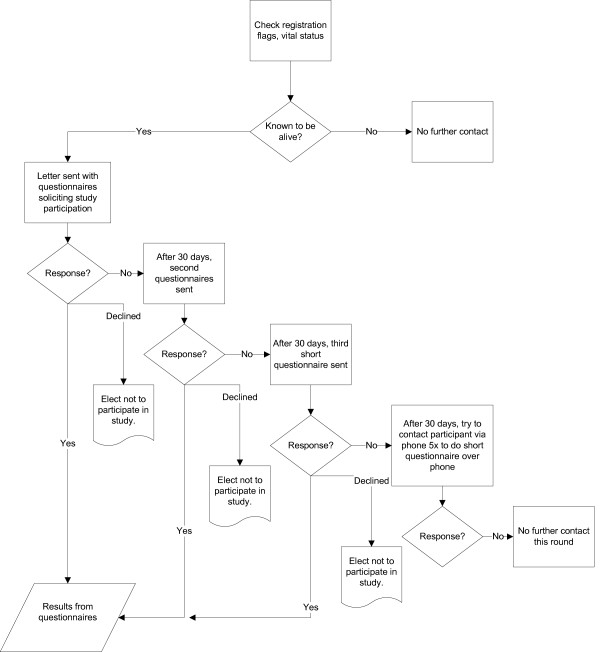
Flow chart showing contact procedures for study participants in The Olmsted County Study of Urinary Symptoms and Health Status among Men, Rochester, Minnesota (1989–2003).

The cohort has been actively followed biennially, with a similar protocol to the initial examination. In follow-up, questionnaires were mailed to participants rather than completed at in-home visits. Our study developed detailed algorithms regarding contact procedures for study participants (Figure 1). Initially, clinic registration and vital status were reviewed. Men who had died or who withdrew from the study in writing were removed from the contact list. However, we examined possible bias due to death by including deaths between contacts as dropouts in ancillary analyses. Letters, a brochure describing the study with a list of study publications, and questionnaires were mailed to study participants on a biennial basis using a 30-day contact interval for non-responders. If participants failed to respond after the third mailing, study personnel attempted to reach them via phone. If contact was not initiated after five phone calls, no further contact was attempted for that round. Any participant could elect not to participate in the study at any time without any negative impact on their medical care at the clinic.

Men who were diagnosed with prostate cancer, received a radical prostatectomy, or transurethral resection of the prostate (TURP) were not excluded from the cohort, but were excluded from participating in the detailed urologic examination. During the second and third biennial follow-up, men who did not participate in the follow-up were replaced by men randomly selected from the community, after being screened for the exclusion criteria used at baseline (n = 229 and n = 103, respectively). Of the replacement men, randomly selected individuals were added to the clinic sub-sample in the years two and four of follow-up, (n = 100 and n = 58, respectively). Since that time the study has been maintained as a closed cohort. At the twelfth year of follow-up, 682 (30.3% of live enrollees) men had dropped out of the study.

### Statistical analysis

Baseline urologic health status, comorbidities, HRQL, and socioeconomic status were compared among the men who dropped out and those who continued to participate in the twelfth year of follow-up of the study. Men who died between contacts (n = 195) were removed from the analysis, including 46 men in the clinic subset. An alpha of p < 0.05 was considered statistically significant.

Urologic measures were dichotomized according to standard cut points. Urinary symptoms were assessed by the AUASI. Scores ranged from 0 (none) to 35 (severe), with scores from 8 to 19 classified as "moderate" symptoms and scores ≥ 20 classified as "severe" symptoms. These two categories were combined in the analyses, and scores > 7 were considered moderate/severe symptoms [16]. Other urinary outcomes evaluated were peak urinary flow rate (< 12 ml/second) [17], prostate volume (> 30 cm^3^) [17], serum PSA level (> 1.4 ng/mL, upper 25^th ^percentile for this cohort at baseline), and creatinine (> 1.4 ng/dL) [18]. A chi-square test was used to compare the distribution of urologic measures among participants and those who dropped out.

Logistic regression was used to calculate age-adjusted odds ratios (OR) comparing individuals who dropped out and those who continued to participate in the twelfth year of follow-up of the study. A multivariable model was constructed to simultaneously adjust for age, urologic health status, comorbidities, HRQL, and socioeconomic factors in predicting dropout from the study. This model contained the variable with the strongest age-adjusted association from each domain. Forward selection logistic regression verified the final model. All 2-way interactions between variables were also evaluated. To account for multiple comparisons in these models a p-value of 0.005 was used for inclusion-exclusion in the model. All analyses were conducted using SAS, version 8.2 (SAS Institute, Cary, N.C.)

## Results

In univariate analyses, we found that older men were more likely to drop out of our study compared to younger men (Table 1). Although men who were 70 years or older at baseline made up the smallest number of participants in the total cohort, this group had the largest dropout. Furthermore, men who dropped out were more likely to have moderate/severe symptoms and decreased urinary flow rates compared to those who remained in the study (Table 1). The presence of an enlarged prostate and elevated PSA did not differ significantly between men who participated and those who dropped out.

**Table 1:** Characteristics of men who participated versus those who dropped out of The Olmsted County Study of Urinary Symptoms and Health Status among Men, Rochester, Minnesota (1989–2003)

	**Baseline Questionnaire†**				
	
	**Participated**		**Dropped out‡**		
	
	**No.**	**%**	**No.**	**%**	**Chi-sq p**
	
AGE (years)					
40–49	734	46.7	336	49.3	0.01
50–59	458	29.2	138	20.2	
60–69	293	18.7	105	15.4	
70+	85	5.4	103	15.1	
AUASI*					
≤ 7	1105	70.4	452	66.3	0.05
> 7	465	29.6	230	33.7	
QMAX (mL/s)*					
≥ 12	1297	82.8	532	78.2	0.01
< 12	269	17.2	148	21.8	
PSA (ng/mL)*					
≤ 1.4	329	75.3	107	70.9	0.28
> 1.4	108	24.7	44	29.1	
VOL (cm^3^)*					
≤ 30	300	68.8	94	62.7	0.17
> 30	136	31.2	56	37.3	

* AUASI, American Urological Association Symptom Index; QMAX, peak urinary flow rate; PSA, Prostate-Specific Antigen; VOL, prostate volume.

† Age was calculated based on the age at baseline questionnaire completion.

‡ No questionnaire completed in seventh biennial contact year.

§ Men who died between contacts were excluded, n = 195.

¶ Of the 195 excluded men, 46 were men in the clinic subset.

# PSA and VOL measurements are from the randomly selected in-clinic subset, n = 588.

We next adjusted for age and determined whether other specific participant characteristics were associated with dropping out of our study (Table 2). Clinical urologic measures were not strongly associated with increased dropout. Men who reported they had ever suffered a stroke were over three times more likely to drop out compared to men who had not suffered a stroke (Table 2). However, this represented a small percentage of the cohort, with only 22 men who suffered a stroke dropping out. Men with any comorbidity present were more likely to drop out of the study than men without any comorbidity. Other factors that were significantly associated with study dropout included presence of ED and dissatisfaction with their urinary condition or overall health. Men who were satisfied with their urinary condition and overall health were less likely to drop out (Table 2). In addition, men who had more interference with living activities from their urinary problems were more likely to drop out than those men with less interference. Lower socioeconomic status was also associated with study dropout. Men who had a high school education were less likely to drop out of the study compared to men without at least a high school education (Table 2). Men reporting higher salaries were also less likely to drop out of the study. Finally, men who were randomly selected to participate in the clinic subset were less likely to drop out than men who were not.

**Table 2:** Age-adjusted comparisons of relative odds of dropping out of The Olmsted County Study of Urinary Symptoms and Health Status among Men based on baseline measurements, Rochester, Minnesota (1989–2003)

**Primary Study Outcomes**	Age-adjusted OR* (95% CI*)
AUASI* (> 7 vs. ≤ 7)	1.19 (0.97, 1.45)
Peak Urinary Flow Rate (QMAX) (< 12 vs. ≥ 12 mL/s)	1.18 (0.93, 1.50)
Prostate Volume (> 30 vs. ≤ 30 cm^3^)	0.98 (0.62, 1.56)
Prostate-Specific Antigen (> 1.4 vs. ≤ 1.4 ng/mL)	0.90 (0.55, 1.46)
Any Benign Prostatic Hyperplasia (Yes vs. No)	1.12 (0.93, 1.36)
Member of Clinic Subset (Yes vs. No)	0.73 (0.59, 0.90)
	
**Comorbidities**	
	
Diabetes (Yes vs. No)	1.24 (0.77, 1.99)
Stroke (Yes vs. No)	3.07 (1.49, 6.33)
Hypertension (Yes vs. No)	1.23 (0.97, 1.56)
Coronary Disease (Yes vs. No)	1.25 (0.92, 1.68)
Any Comorbidity (Yes vs. No)	1.31 (1.06, 1.61)
	
**Other Urologic Measures**	
	
Creatinine (> 1.4 vs. ≤ 1.4 ng/dL)	1.10 (0.45, 2.66)
Erectile Dysfunction (Yes vs. No)	1.69 (1.26, 2.28)
	
**Quality of Life Measures**	
	
Satisfied with Urinary Condition (Yes vs. No)	0.65 (0.49, 0.87)
Satisfied with overall health/HRQL* (Yes vs. No)	0.29 (0.19, 0.43)
Visited a doctor more than 4 times in the past 12 months (Yes vs. No)	1.30 (0.96, 1.75)
Interference Score (> 9 vs. ≤ 9)	1.32 (1.07, 1.62)
	
**Socioeconomic Measures**	
	
High School Graduate or More (Yes vs. No)	0.40 (0.29, 0.56)
Married or Living Together (Yes vs. No)	0.81 (0.60, 1.08)
Salary ($15,000–$34,999 vs. ≤ $14,999/year)	0.65 (0.48, 0.90)
Salary (≥ $35,000 vs. ≤ $14,999/year)	0.44 (0.33, 0.60)

* OR, Odds Ratio; CI, Confidence Interval; AUASI, American Urological Association Symptom Index; HRQL, Health-Related Quality of Life.

When considered simultaneously in a multivariable model, older men with poor health status were more likely to dropout; however the magnitude of the associations decreased. Men with a good health related quality of life score, more education and participation in the clinic subset were more likely to remain in the study (Table 3).

**Table 3:** Odds of dropping out of The Olmsted County Study of Urinary Symptoms and Health Status among Men before the seventh biennial contact based on the multivariable model baseline measurements, Rochester, Minnesota (1989–2003)

**Variable**	Unadjusted OR* (95% CI*)	**Multivariable Adjusted OR (95% CI)**
Baseline age 40–49	1.00 (Reference)	1.00 (Reference)
Baseline age 50–59	0.66 (0.52, 0.83)	0.59 (0.46, 0.74)
Baseline age 60–69	0.78 (0.61, 1.01)	0.56 (0.42, 0.75)
Baseline age 70+	2.65 (1.93, 3.63)	1.49 (1.01, 2.19)
Erectile Dysfunction	1.86 (1.44, 2.41)	1.52 (1.11, 2.07)
Stroke	3.99 (2.00, 7.97)	2.39 (1.13, 5.05)
Satisfied with overall health/HRQL*	0.28 (0.18, 0.41)	0.35 (0.23, 0.55)
High School Graduate or more	0.38 (0.28, 0.51)	0.43 (0.30, 0.60)
Member of Clinic Subset	0.79 (0.65, 0.97)	0.74 (0.60, 0.92)

* OR, Odds Ratio; CI, Confidence Interval; HRQL, Health-Related Quality of Life.

## Discussion

Our results suggest several factors that may help predict which men are more likely to drop out versus continue to participate in a long term study of urologic health. A critical finding however, is that, after adjusting for age, there were no differences in primary study outcomes at baseline between individuals who dropped out and those who continued to participate in the twelfth year of follow-up. Our findings showed that men with ED were more likely to drop out of the study compared to those without ED. It is possible that this condition may have been too frustrating for the men to continue to participate in the study or initially they may have perceived a potential benefit for this condition by participating in the study. Alternatively, as ED is associated with several comorbidities and life-style factors, i.e. smoking, diabetes, cardiovascular disease [19,20], drop out associated with ED may be related to other health issues.

Our results show that older men with poorer general health status were more likely to drop out of the study. The increased dropout in older men with comorbid conditions is consistent with other studies [21-23], where nonresponse bias in the older subset was a concern. Therefore, additional efforts may be required to encourage older participants and those with co-morbid conditions to continue participation in long-term studies. In addition, this demonstrates the need for assessing the effects of age stratification and age-adjustment when evaluating study outcome variables. Furthermore, comorbidities may be confounded with age, as the risk for comorbid conditions tends to increase with age [21-23].

Health-related quality of life measures also showed that men who suffered more interference from their urologic symptoms were more likely to drop out than men with less interference. This is consistent with a five-year natural history study of BPH in Scotland [2] in which men who reported having the highest levels of bother and interference at baseline were less likely to participate in the survey at 5 years. It was proposed that one reason for this was that men may adjust to their symptoms over time and adapt their life-style to accommodate symptoms. Alternatively, these men may not have seen any personal benefit to continued participation in the study.

Men with a lower socioeconomic status were also more likely to drop out of the study. Although few urologic studies have documented the effect of education level on study dropout, numerous studies of other disease conditions have documented the potential effect of education level on study attrition. For example, in a longitudinal, multicenter AIDS cohort study examining retention after 9.5 years, lower education level was significantly associated with nonparticipation [24]. In addition, a study examining predictors of inactivation and reasons for participant inactivation during skin cancer chemoprevention found that a low education level was a significant predictor of inactivation [25]. One explanation for this finding is that men with a lower education level may not fully appreciate the benefits of study participation. An approach to this issue may be to offer educational seminars or orientation sessions for study subjects that outline and discuss the benefits participants and society may receive from the study.

In our study, with the approval of the Mayo IRB, we have used a combination of active and passive follow-up to assess characteristics associated with participant dropout. This has allowed us to gauge the probability and degree of study bias due to nonparticipation. This level of follow-up is unique, and may not be feasible for other studies, as few studies have access to medical records for both participants and nonparticipants. However, our study may have greater validity for the target study population, due to our ability to assess not only participants, but also those subjects who have previously dropped out of the study.

There are some limitations to the generalizability of this study. All participants in the Olmsted County Study of Urinary Symptoms and Health Status among Men are Caucasian and were 40–79 years of age at study entry. Therefore, the ability to generalize these findings to other ethnic populations and age groups may be limited. We excluded men who died between contacts. By doing this, we assumed that death was unrelated to our outcome. However, the men who died may have been in poorer health and may have been at greater risk for clinical urologic conditions. We repeated our initial analysis including deaths between contacts as dropouts and the results were similar (data not shown). Moreover, nonparticipation, *per se*, was not related to primary study outcomes at baseline (after adjusting for age).

Another potential limitation was the 55% participation rate; however, factors associated with participation may not be the same factors associated with dropout. We have previously documented differences in the characteristics of baseline participants and nonparticipants in age, home location, and prior history of urologic conditions [26]. The age-adjusted period prevalence rate for benign prostatic hyperplasia was 9.6% (95% CI: 8.1, 11.0) for full participants, 8.2% (95% CI: 5.8, 10.6) for partial participants and 5.3% (95% CI: 3.6, 6.9) for complete non-responders. These results suggest that initial participation in our study may have been at least partially driven by concerns about urologic health. No differences were found between participants and nonparticipants in other major medical comorbidities. Additionally, longitudinal assessment of the cohort did not show any differences in urologic outcomes identified through medical record abstraction between participants and nonparticipants [27]. Therefore, while it is possible that some of the men who did not initially participate in our study might have been more likely to drop out, an earlier study of these groups did not suggest that initial nonparticipants differed dramatically from participants.

## Conclusion

Regardless of these limitations, this study demonstrates that cohort studies may encounter differential attrition associated with poor health status, increasing age, lower education level and socioeconomic status. Even well designed cohort studies and clinical trials will suffer if differential attrition biases results. Thorough planning, special efforts to encourage continued participation and statistical methods can be implemented to minimize dropout and correct for non-response bias. Differential attrition in this study did not bias our primary study outcomes, however future reports from this and other urologic studies which focus on ED will need to address the degree to which dropout might bias those results.

## Competing interests

The author(s) declare that they have no competing interests.

## Authors' contributions

NMG and DJJ wrote and revised the paper. MEM did the data analysis, assisted with data collection coordination, and reviewed manuscript. MML assisted with the initiation of the study project. ROR assisted with initiation of study project. CJG advised on data analysis and critically reviewed manuscript. SJJ had study concept and design, contributed to writing and revising manuscript.

## Pre-publication history

The pre-publication history for this paper can be accessed here:


